# Probing astrocyte metabolism *in vivo*: proton magnetic resonance spectroscopy in the injured and aging brain

**DOI:** 10.3389/fnagi.2015.00202

**Published:** 2015-10-28

**Authors:** Janna L. Harris, In-Young Choi, William M. Brooks

**Affiliations:** ^1^Hoglund Brain Imaging Center, University of Kansas Medical CenterKansas City, KS, USA; ^2^Department of Anatomy and Cell Biology, University of Kansas Medical CenterKansas City, KS, USA; ^3^Department of Neurology, University of Kansas Medical CenterKansas City, KS, USA; ^4^Department of Molecular and Integrative Physiology, University of Kansas Medical CenterKansas City, KS, USA

**Keywords:** proton magnetic resonance spectroscopy, astrocytes, traumatic brain injury (TBI), aging, myo-inositol, glutathione, glutamic acid, lactic acid

## Abstract

Following a brain injury, the mobilization of reactive astrocytes is part of a complex neuroinflammatory response that may have both harmful and beneficial effects. There is also evidence that astrocytes progressively accumulate in the normal aging brain, increasing in both number and size. These astrocyte changes in normal brain aging may, in the event of an injury, contribute to the exacerbated injury response and poorer outcomes observed in older traumatic brain injury (TBI) survivors. Here we present our view that proton magnetic resonance spectroscopy (^1^H-MRS), a neuroimaging approach that probes brain metabolism within a defined region of interest, is a promising technique that may provide insight into astrocyte metabolic changes in the injured and aging brain *in vivo*. Although ^1^H-MRS does not specifically differentiate between cell types, it quantifies certain metabolites that are highly enriched in astrocytes (e.g., *Myo*-inositol, mlns), or that are involved in metabolic shuttling between astrocytes and neurons (e.g., glutamate and glutamine). Here we focus on metabolites detectable by ^1^H-MRS that may serve as markers of astrocyte metabolic status. We review the physiological roles of these metabolites, discuss recent ^1^H-MRS findings in the injured and aging brain, and describe how an astrocyte metabolite profile approach might be useful in clinical medicine and clinical trials.

## Introduction

Once thought to be merely part of the “glue” that holds together the neurons in the central nervous system (CNS), astrocytes are now known to serve a variety of complex and essential roles in CNS function. Astrocytes are intimately involved in regulating neurotransmission and local cerebral blood flow, and in maintaining the osmotic, pH, and neurotransmitter homeostasis of the extracellular environment in a manner that is essential for normal synaptic function. They influence neuronal structure and function by releasing growth factors and cytokines, and they play a critical role in brain energy metabolism, taking up glucose from the blood and furnishing energy metabolites to neurons (Sofroniew and Vinters, 2010; Allaman et al., 2011).

After a brain injury, molecular signals induce astrocyte activation and migration to the site of injury. These reactive astrocytes play two somewhat conflicting roles. One is protective, by controling extracellular ionic and neurotransmitter balance, providing essential metabolic substrates to neurons, scavenging oxygen free radicals, and contributing to the formation of an astroglial scar which protects the adjacent, relatively uninjured tissue surrounding the brain lesion (Chen and Swanson, 2003). The other may exacerbate injury pathology by playing a key role in the neuroinflammatory response.

Astrocytes also progressively accumulate in the brain during normal aging, increasing in both number and size (Cotrina and Nedergaard, 2002). These aging-related astrocyte changes might, in the event of an injury, contribute to the exacerbated injury response and poorer outcomes observed in older traumatic brain injury (TBI) patients (Popa-Wagner et al., 2011).

**Figure 1:**
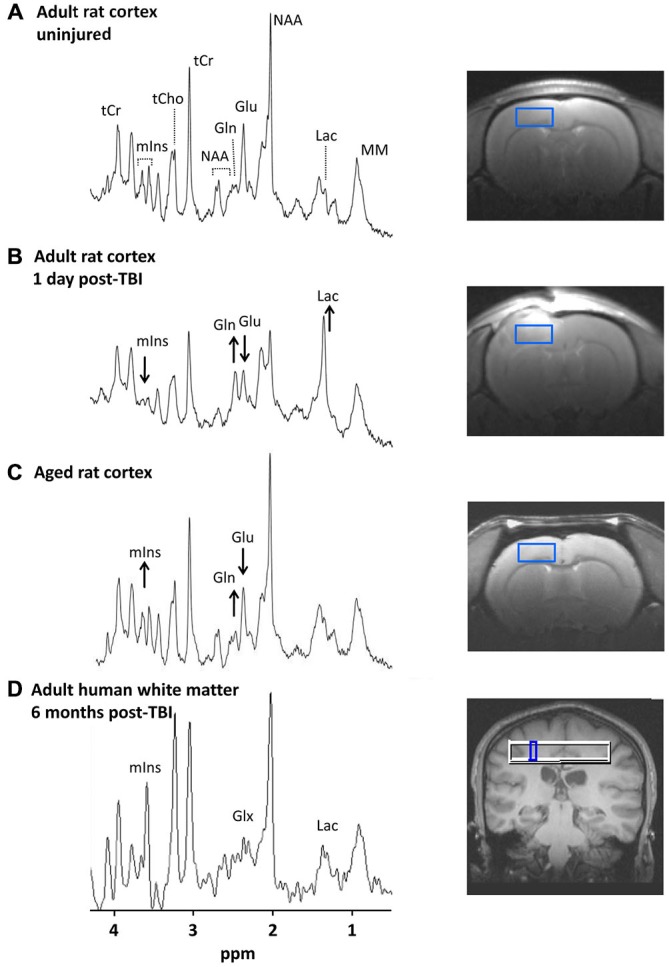
**^1^H-magnetic resonance spectra in the injured and aging brain. (A–C)** show spectra from rat cortex (2.7 × 1.3 × 2.7 mm^3^ ROI). Images to the right show the location of each ROI. **(A)** Spectrum from an un-injured adult male rat (3 months old). Major metabolite peaks visible at 9.4 T are labeled. **(B)** Spectrum from the same animal 1 day after a moderate severity controlled cortical impact TBI. Acute post-injury changes are visible including lower mIns and Glu and higher Gln and Lac. **(C)** Spectrum from an un-injured aged rat (22 months old). More subtle metabolic changes in the aging rat brain compared with younger controls include lower Glu and higher mIns and Gln. The complex resonance pattern of GSH is not immediately visible even at high magnetic field strengths but is detectable through digital signal processing. **(D)** Spectrum from human white matter (5 × 5 × 15 mm^3^ ROI) of an adult male (20 years old) at 6 months post-TBI. The mIns, Lac, and Glx peaks visible at 3 T are indicated. Human studies have reported elevated mIns and Glx in TBI survivors from sub-acute to chronic time points (~1 week to 6 months post-injury; Brooks et al., 2000; Ashwal et al., 2004a,b; Kierans et al., 2014). Abbreviations: ROI, region of interest; TBI, traumatic brain injury; mIns, *myo*-inositol; Glu, glutamate; Gln, glutamine; Lac, lactate, Glx, Glutamate + Glutamine; NAA, N = acetylaspartate; tCr, total creatine; tCho, total choline; MM, macromolecules. Figure reproduced in part from Harris et al. (2012) and Harris et al. (2014).

Given these critical roles of astrocytes in the function of the normal and injured brain, the ability to monitor key aspects of astrocyte metabolic status in the intact, living organism would be of great value. We propose that proton magnetic resonance spectroscopy (^1^H-MRS) provides this capability. ^1^H-MRS is a non-invasive imaging technique that quantifies specific brain metabolites within a defined brain region of interest (ROI; Figure 1). Such metabolites can serve as biomarkers of specific molecular and cellular mechanisms, which can be used to monitor brain pathologies and effectiveness of treatment. ^1^H-MRS can be carried out on most magnetic resonance imaging (MRI) scanners, and can quantify from 3 to 20 or more metabolites *in vivo* depending on the magnetic field strength and the selected pulse sequences. These metabolites reflect various physiological processes including bioenergetics, oxidative stress, neurotransmission, and neuroinflammation. The high reproducibility of ^1^H-MRS data acquired *in vivo* has been demonstrated in humans and rodents (Brooks et al., 1999; Harris et al., 2012).

In this article, we advance the view that ^1^H-MRS is a promising technique for non-invasive monitoring of astrocyte metabolism *in vivo*. Although ^1^H-MRS does not specifically differentiate between cell types, it can quantify metabolites that are enriched in astrocytes or are related to astrocyte-neuron metabolic shuttling. We identify five ^1^H-MRS-detectable metabolites related to astrocyte metabolic status (Table 1), review the known physiological roles of these compounds, discuss recent ^1^H-MRS findings in the injured and aging brain and describe how an astrocyte metabolite profile approach might be useful in clinical medicine and clinical trials.

**Table 1:** **Summary of changes in astrocyte-relevant metabolites seen with ^1^H-MRS *in vivo***.

	Aging	TBI	Aging References	TBI References
mIns	↑	↓ (early), ↑ (later)	Chang et al. (1996), Reyngoudt et al. (2012), Zhang et al. (2013) and Harris et al. (2014)	Schuhmann et al. (2003), Ashwal et al. (2004a,b), Xu et al. (2011), Harris et al. (2012) and Kierans et al. (2014)
GSH	↓	↓	Emir et al. (2011)	Harris et al. (2012)
Gln	↑	↑	Kaiser et al. (2005), Duarte et al. (2014) and Harris et al. (2014)	Xu et al. (2011) and Harris et al. (2012)
Glu	↓	↓	Kaiser et al. (2005), Duarte et al. (2014) and Harris et al. (2014)	Schuhmann et al. (2003), Xu et al. (2011) and Harris et al. (2012)
Lac	~	↑	Urrila et al. (2004), Paban et al. (2010) and Harris et al. (2014)	Ross et al. (1998), Ashwal et al. (2000), Schuhmann et al. (2003), Lescot et al. (2010) and Harris et al. (2012)

*Symbols and abbreviations: ↑ = increase, ↓ = decrease, ~ = no change or mixed results, mIns = *myo*-inositol, GSH = glutathione, Gln = glutamine, Glu = glutamate, Lac = lactate*.

## Components of the Astrocyte Metabolite Profile

### Myo-inositol

*Myo*-inositol (mIns) is the most abundant isomer of inositol in animal tissues. mIns is a component of membrane phospholipids, plays a role in intracellular second messenger cascades, and is an important organic osmolyte. Because the concentration of mIns is higher in astrocytes than in neurons (Brand et al., 1993), mIns has widely been considered an astroglial marker. Due to its high concentration in brain ranging from 4–10 μmol/g, the mIns peak is readily detectable with *in vivo* ^1^H-MRS in both laboratory animals (≥3 Tesla, T) and human subjects (≥1.5 T; Kreis et al., 1992; Pfeuffer et al., 1999; Blüml, 2013).

mIns plays an important role in osmotic control of astrocyte cell volume. Decreased mIns is observed early after TBI (Schuhmann et al., 2003; Xu et al., 2011; Harris et al., 2012; Figure 1B), which may reflect mIns efflux from astrocytes as a volume-regulatory strategy under conditions of edema, or alternatively may reflect cell lysis and death (Zhao et al., 2003). By contrast, pathologically activated astrocytes with larger cell volumes tend to have increased mIns (Chang et al., 2013). Thus, later (beginning at several days to weeks) in the progression of the injury, increased mIns suggesting neuroinflammation and reactive astrogliosis has been observed (Bitsch et al., 1999; Schuhmann et al., 2003; Ashwal et al., 2004b; Filibian et al., 2012; Harris et al., 2012; Kierans et al., 2014).

mIns levels also increase with normal brain aging in both humans and animal models (Chang et al., 1996; Reyngoudt et al., 2012; Zhang et al., 2013; Harris et al., 2014; Figure 1C), suggesting an aging-related increase in activated astrocytes. Therefore ^1^H-MRS might be useful to monitor the process of “inflamm-aging”, the low-grade, chronic up-regulation of pro-inflammatory signals in the normal aging brain (Franceschi et al., 2007).

A link between increased mIns and astrocyte activation is supported by invasive studies in animal models. In rats in status epilepticus, hippocampal mIns was strongly correlated with staining for two astrocyte markers, glial fibrillary acidic protein (GFAP) and S100b (Filibian et al., 2012). In models of Alzheimer’s disease and TBI, increased mIns corresponded with increased GFAP staining within the same ROI (Chen et al., 2009) and following the same time course (Schuhmann et al., 2003). Moreover, an experimental anti-inflammatory treatment in a mouse model of Alzheimer’s disease attenuated both the increase in ^1^H-MRS-detectable mIns and the increase in GFAP-positive astrocytes (Chen et al., 2012).

Data from humans are more limited, but also support a link between mIns and astrocyte activation. In patients with multiple sclerosis, GFAP-positive biopsies of demyelinating lesions corresponded with elevated mIns measured in the same location (Bitsch et al., 1999). In patients with schizophrenia, mIns concentrations in the amygdala correlated with serum levels of S100b (Rothermundt et al., 2007). A group of high-likelihood Alzheimer’s disease patients had higher mIns and higher postmortem GFAP staining than low-likelihood controls, although in the combined sample GFAP and mIns levels did not reach a statistical correlation (Murray et al., 2014).

In sum, the evidence from ^1^H-MRS suggests that the mIns changes observed in brain injury and aging reflect physiological changes in astrocytes. As others have recently suggested (Rae, 2014), mIns is unlikely to be a marker for astrocyte cell density alone since it is evident that pathological conditions such as osmotic stress can alter intracellular concentrations of mIns (Strange et al., 1994). Rather, we propose that early decreases after a brain injury might reflect edema and/or cell lysis. Increases seen in aging or at later intervals after a brain injury likely reflect astrocyte activation and proliferation during neuroinflammation.

### Glutathione

The reduced form of glutathione (GSH) and ascorbate (Asc, or vitamin C) are the two most prominent endogenous antioxidants in the CNS, protecting cells from damaging reactive oxygen species and preserving essential cellular functions. While Asc predominates in neurons, GSH concentrations are considerably higher in astrocytes (Rice and Russo-Menna, 1998; Sun et al., 2006). Therefore, GSH measured with ^1^H-MRS may provide an *in vivo* marker of astrocyte antioxidant status. Additional cellular functions of GSH include amino acid transport, acting as a storage form of cysteine and a cofactor for redox reactions, and protecting neuronal signal transduction (Brown, 1994; Rae, 2014). GSH is routinely quantified in animal studies using ^1^H-MRS at high magnetic fields, but is somewhat more challenging to measure in humans, requiring specialized acquisition strategies (Trabesinger and Boesiger, 2001; Terpstra et al., 2003; Choi et al., 2011).

Brain GSH levels fall rapidly after TBI (Ansari et al., 2008a,b; Harris et al., 2012), consistent with an early post-injury increase in reactive oxygen species that depletes brain antioxidant reserves (Kontos and Povlishock, 1986; Hall et al., 2010). Moreover, the depletion of GSH is related to the severity of brain damage (Harris et al., 2012; Di Pietro et al., 2014). In contrast, GSH levels may increase in pathologies where astrocytes are chronically activated and recruited. In a rat model of epilepsy, Filibian et al. (2012) showed that elevated GSH concentrations in the hippocampus were highly correlated with quantitative GFAP staining, supporting the use of GSH as an *in vivo* marker of astrocyte activation.

Recent evidence from animal models and humans points to lower GSH concentrations in the aging brain (Maher, 2005; Emir et al., 2011). Our group has found that the regional pattern of GSH depletion in the aging brain differs somewhat from that of Asc, suggesting that local populations of astrocytes and neurons might be differentially sensitive to oxidative stress during aging (Harris et al., 2014). In any case, lower antioxidant levels suggest that the brain’s ability to combat oxidative stress may be impaired in aging. Lower GSH levels could contribute to an age-related decline in cellular function and increase the brain’s susceptibility to insult (Maher, 2005). This notion is supported by studies of brain injury in aged rats, which show more severe oxidative damage after TBI compared with young adult animals (Shao et al., 2006; Gilmer et al., 2010). Since antioxidant therapies are currently under investigation for both brain trauma and aging, GSH offers a potential *in vivo* marker to evaluate therapeutic target engagement.

### Glutamate and glutamine

Glutamate (Glu) serves as the major excitatory neurotransmitter and is a precursor of γ-aminobutyric acid (GABA), the major inhibitory neurotransmitter in the CNS. Glu is also closely associated with glutamine (Gln) via the Glu-Gln cycle between neurons and astrocytes. After its release from neuronal synapses, Glu is taken up by nearby astrocytes, converted to Gln, then transported back to neurons. Overall, brain Glu concentrations range from 6–13 μmol/g and Gln from 2–4 μmol/g (Michaelis et al., 1993; Petroff et al., 1995; Hurd et al., 2004). Although Glu is found in all cells, glutamatergic neurons contain the highest levels of Glu compared with other neuronal and glial cell types. In contrast, because Gln synthetase is exclusive to astrocytes, Gln levels are typically much higher in astrocytes. Therefore, ^1^H-MRS measures of the relative concentrations of Gln and Glu may be useful to infer changes in astrocyte populations or metabolic status.

Studies by our group and others in animal models point to rapidly increased Gln concurrent with decreased Glu after brain injury (Lei et al., 2009; Xu et al., 2011; Harris et al., 2012; Figure 1B), a finding consistent with injury-induced Glu release from neurons followed by rapid uptake and conversion to Gln by astrocytes i.e., the Glu-Gln cycle (Bartnik-Olson et al., 2013). This likely reflects a critical role of astrocytes in helping to limit the effects of Glu excitotoxicity in the acute post-injury period.

In human and animal studies of the normal aging brain, the Gln/Glu ratio increases progressively (Kaiser et al., 2005; Duarte et al., 2014; Harris et al., 2014; Figure 1C), suggesting a potential aging-related shift in the Glu-Gln cycle. This is consistent with the finding of ~30% lower flux through the Glu-Gln cycle in healthy elderly humans compared with younger adults (Boumezbeur et al., 2010). Whether such changes in neuronal-astrocyte metabolic shuttling are responsible for age-related declines in cognitive function remains an open question. However, this could be addressed by studies that link ^1^H-MRS measures of Glu and Gln with cognitive function in aging.

### Lactate

According to the lactate shuttle hypothesis, glycolysis in astrocytes generates lactate (Lac), which is then released from the astrocytes to serve as a preferred fuel for neurons (Pellerin and Magistretti, 1994). Lac is present in brain at approximately 0.5 μmol/g, which is approaching the lower threshold for detection in animals and humans with standard ^1^H-MRS techniques. However, in pathological conditions that alter bioenergetics and result in increased Lac accumulation, the Lac peak is easily detectable.

After a brain injury, an acutely increased energy demand results in increased glycolysis, which along with impaired respiratory function of mitochondria results in rapid Lac accumulation. Elevated brain Lac after injury has been well documented with spectroscopic methods (Ross et al., 1998; Ashwal et al., 2000; Schuhmann et al., 2003; Lescot et al., 2010; Harris et al., 2012; Figure 1B) and microdialysis (Nilsson et al., 1990; Yokobori et al., 2011). Since Lac is produced by astrocytes in areas of glutamatergic neuronal activity, astrocytes are likely a major source of this Lac spike early after TBI. In support of this view, Schuhmann et al. (2003) reported an increase in Lac up to 24 h after TBI in animals which corresponded to an increase in glutamate dehydrogenase (a Glu metabolizing enzyme preferentially localized in astrocytes) in the ROI used for ^1^H-MRS. Since Lac has been shown to strongly predict poor outcomes after TBI in humans (Ashwal et al., 2000; Glenn et al., 2003), the ability to track Lac accumulation *in vivo* could have important clinical applications.

In contrast, reports on Lac concentrations in normal brain aging are inconsistent, ranging from falls to increases (Urrila et al., 2004; Paban et al., 2010; Emir et al., 2011; Zhang et al., 2013; Duarte et al., 2014; Harris et al., 2014), perhaps reflecting a varying capacity for bioenergetic homeostasis in individual subjects or brain regions. Altered Lac levels in the aging brain compared with younger adults implies a mismatch between astrocytic glycolysis and neuronal oxidative metabolism. If metabolic coupling between neurons and astrocytes is indeed altered in aging, this could impair the ability of the brain to respond to insult and might contribute to the poor outcomes seen after TBI in older individuals.

## Challenges for Magnetic Resonance Spectroscopy?

We envision a future in which ^1^H-MRS biomarkers will aid in prognostication and in selecting therapies to mitigate the cognitive impairment seen with TBI, or the cognitive decline seen with aging. Furthermore, these biomarkers will provide a non-invasive means to track therapeutic target engagement and efficacy. Although at present we can acquire signals from numerous ^1^H-MRS biomarkers in animals, there remains considerable opportunity for developing new acquisition approaches to expand the repertoire of metabolites routinely quantified in humans.

Using widely available spectroscopic acquisitions, Lac and mIns are easily quantified in animal models and in humans. However, GSH, Glu, and Gln are more challenging, largely due to overlapping signals. The issue of overlapping signals can be resolved at higher magnetic field strengths; indeed, there is already a proliferation of MRI scanners at 7 T and higher for research use and some are advocating the use of fields greater than 3 T for clinical investigation. An alternative approach is to develop more sophisticated acquisition sequences such as two-dimensional spectroscopy (Lin et al., 2015) and sequences aimed at individual brain metabolites such as double quantum acquisitions for GSH (Choi et al., 2011).

In addition to technical challenges of data acquisition, challenges of data validation remain to be addressed before ^1^H-MRS biomarkers can be used in clinical applications. The precise mechanisms underlying altered mIns, GSH, Gln/Glu, and Lac following brain injury or in aging are yet to be clarified. Thus, the use of these metabolites as biomarkers of astrocyte metabolic status needs to be further validated specifically in the context of brain injury and aging, by comparing their levels with other quantitative measures of astrocyte physiology. In addition, there is currently little information available about the possible contribution of other CNS cell types such as microglia to the ^1^H-MRS profiles. Non-invasive ^1^H-MRS approaches in animal models are well suited to addressing these questions, since they permit intra-subject validation of pathological mechanisms when *in vivo* scanning is followed by invasive histological and biochemical analyses of the same tissue. Once validated in animal models, ^1^H-MRS can form the basis for translational imaging studies in humans, where it can be combined with measures of cognitive function and other clinically relevant outcome measures.

## How Would an Astrocyte Metabolite Profile be Useful?

We have identified five ^1^H-MRS-detectable metabolites related to astrocyte metabolic status. Although taken individually, each metabolite might provide valuable information on individual molecular or cellular mechanisms as discussed above, they can also be considered in combination—as an astrocyte metabolite profile. On the whole, the profiles of astrocyte-relevant metabolite changes in brain aging and injury look remarkably similar (Table 1) and are consistent with two possible pathological mechanisms associated with aging and injury. The first is *bioenergetic disruption*. Impaired mitochondrial respiration is known to increase Lac accumulation and may be related to increased Gln/Glu ratio in the aging and injured brain since Glu is derived from α-ketoglutarate, a TCA cycle intermediate. Moreover, bioenergetic deficit can also result in reduced production of endogenous anti-oxidants, including GSH. Disrupted mitochondrial respiration can also cause increased reactive oxidative species, which would further consume GSH. A second pathological mechanism is inflammation, associated with *astrocyte activation and recruitment*, which has also been reported in both aging and injured brain. An increased astrocyte pool would result in elevated mIns and could also yield elevated Gln/Glu.

Several lines of evidence suggest that a well-characterized metabolite profile is likely to be useful as an overall biomarker of injury severity and for prognostication, for stratifying patients into trials, and for treatment selection and management.

Larger metabolite changes are associated with greater injury severity and worse functional outcomes. For example, we have shown in an animal model of TBI that the magnitude of change in ^1^H-MRS-detectable metabolites is greater in tissue located closer to the primary lesion site (Harris et al., 2012). Further data from our laboratory show that Lac, GSH, and Glu, key components of the astrocyte metabolite profile, measured within a day of injury predicted the amount of subsequent tissue loss at 2 weeks (Sharrock et al., 2015). In human TBI survivors, Ashwal *et al*. found that greater Lac and mIns changes were associated with poorer Glasgow coma scores and also with poorer outcome (Ashwal et al., 1997, 2004b). Thus, the astrocyte metabolite profile shows promise as a prognostic indicator for brain injury.

The astrocyte metabolite profile might also be useful in understanding cognitive decline in aging. As shown in Table 1, the metabolite profile of the aging brain is similar to that of a younger brain following TBI. The TBI profile is known to be associated with poorer cognitive functioning (Ashwal et al., 1997, 2004b). Thus, we might speculate that if this profile is seen in aging, it could provide information related to declining cognitive function that could be used to identify individuals at risk for pathologies of aging or to stratify participants for trials.

Finally, astrocyte-associated ^1^H-MRS biomarkers might be used for treatment selection. To the extent that ^1^H-MRS-detected metabolites are associated with cellular and molecular mechanisms, changes observed in prognostic studies might identify targets for therapeutic intervention. For example, an elderly patient with cognitive impairment and lower GSH might benefit from anti-oxidant therapy and a TBI patient with high mIns might benefit from anti-inflammatory treatment. Finally, these biomarkers could be used as pharmacodynamic indicators to verify target engagement in the brain, and to help confirm treatment efficacy.

## Conclusions and Future Directions

^1^H-MRS is one of the few techniques that can non-invasively assess the chemical concentrations and metabolic pathways of the brain in living individuals. We propose that ^1^H-MRS-detectable metabolites can serve as translational biomarkers of certain aspects of brain health and pathology. Specifically, certain metabolites seen on ^1^H-MRS promise insight into the metabolic status of astrocytes *in vivo*.

The studies we have discussed here have largely focused on ^1^H-MRS findings in normal brain aging *or* brain injury. Although it is clear that the risk of brain injury is elevated in older individuals and that outcomes in these patients are significantly worse (Thompson et al., 2006), there is a dearth of information on the combined effects of brain aging *and* brain injury. Thus, there is a critical need for further studies aimed at better understanding the effects of aging on TBI pathophysiology.

^1^H-MRS provides the ability to monitor specific aspects of brain metabolic status in animals and humans as well as across different biological conditions. Taken together, the evidence we have presented suggests that ^1^H-MRS provides a unique window onto astrocyte metabolic changes *in vivo*. Given the many essential roles of astrocytes in brain function, we believe that the ability to monitor astrocyte metabolism *in vivo* will provide important insights into pathological changes in the aged and injured brain—a critical step for developing and translating therapies to improve long-term outcomes in this vulnerable patient population.

## Conflict of Interest Statement

The authors declare that the research was conducted in the absence of any commercial or financial relationships that could be construed as a potential conflict of interest.
